# Measuring the timeliness of childhood vaccinations: Using cohort data and routine health records to evaluate quality of immunisation services

**DOI:** 10.1016/j.vaccine.2017.10.085

**Published:** 2017-12-18

**Authors:** Suzanne Walton, Mario Cortina-Borja, Carol Dezateux, Lucy J. Griffiths, Karen Tingay, Ashley Akbari, Amrita Bandyopadhyay, Ronan A. Lyons, Helen Bedford

**Affiliations:** a Population, Policy and Practice Programme, UCL Great Ormond Street Institute of Child Health, London WC1N 1EH, UK; b Farr Institute, Swansea University Medical School, Wales SA2 8PP, UK

**Keywords:** Vaccination, Timeliness, Child, Millennium Cohort Study (MCS), Child health systems

## Abstract

**Background:**

To achieve full benefits of vaccination programmes, high uptake and timely receipt of vaccinations are required.

**Objectives:**

To examine uptake and timeliness of infant and pre-school booster vaccines using cohort study data linked to health records.

**Methods:**

We included 1782 children, born between 2000 and 2001, participating in the Millennium Cohort Study and resident in Wales, whose parents gave consent for linkage to National Community Child Health Database records at the age seven year contact. We examined age at receipt, timeliness of vaccination (early, on-time, delayed, or never), and intervals between vaccine doses, based on the recommended schedule for children at that time, of the following vaccines: primary (diphtheria, tetanus, pertussis (DTP), polio, Meningococcal C (Men C), *Haemophilus influenzae* type b (Hib)); first dose of measles, mumps and rubella (MMR); and pre-school childhood vaccinations (DTP, polio, MMR). We compared parental report with child health recorded MMR vaccination status at age three years.

**Results:**

While 94% of children received the first dose of primary vaccines early or on time, this was lower for subsequent doses (82%, 65% and 88% for second and third doses and pre-school booster respectively). Median intervals between doses exceeded the recommended schedule for all but the first dose with marked variation between children. There was high concordance (97%) between parental reported and child health recorded MMR status.

**Conclusions:**

Routine immunisation records provide useful information on timely receipt of vaccines and can be used to assess the quality of childhood vaccination programmes. Parental report of MMR vaccine status is reliable.

## Highlights

• Most children received the first dose of primary vaccines on time.
• Timeliness of vaccination decreased with vaccine dose.
• Most children had appropriate intervals between doses; marked variation occurred.
• The quality of routine vaccination records in Wales is high.
• Parental report of MMR status is reliable.

## 1 Introduction

To achieve their full benefit, timely delivery of vaccines as well as high uptake are required [1]. The timeliness of vaccinations, that is vaccination at the earliest appropriate age, should be an important public health goal, and yet this information is often lacking as coverage is the usual metric used. Children receiving vaccinations late remain susceptible to vaccine preventable diseases: this may jeopardise their own health, as well as that of younger siblings and compromise herd immunity with consequent potential risk of disease outbreaks. Conversely, vaccines given too early or with a shortened interval between doses may result in a suboptimal immune response, leading to a false sense of protection. Timely immunisation is important to protect against infections with peaks in incidence or particular severity in the very young, for example pertussis, meningococcal B and *Haemophilus influenzae* type b.

Vaccination timeliness has been investigated in the USA, New Zealand, Australia, Belgium, Sweden and low income country settings, but there is a paucity of published research in the UK. In Australia, vaccination delays were more common for later doses and for vaccines given at an older age [2]. In the USA, only a quarter of children received all vaccines according to recommended immunisation schedules [3]. Luman found that timeliness varied significantly by vaccine type: 5–14% of children had received vaccines too early to be considered effective [4], [5]. In Belgium up to 32% of infants experienced delay in receiving the first dose of measles, mumps and rubella (MMR) vaccine and 95% for the third dose of diphtheria, tetanus and pertussis (DTP) vaccine [6].

In the UK, vaccination coverage is reported quarterly and annually for the routine vaccines for children reaching the ages of one, two and five years in the relevant evaluation period [7]. Although valuable for monitoring trends, these data give no insight into whether vaccines were given on time according to the schedule. For example, a fully vaccinated two year old child may actually have been under-vaccinated for a considerable period of that time. High overall coverage has been achieved in the UK, but persisting inequalities leave gaps in immunity. Exploring vaccine timeliness and ensuring timely vaccine delivery may help to address these inequalities.

Previous research based on parental report of immunisation status found high vaccine uptake among participants in the Millennium Cohort Study (MCS) [8], [9], [10]. In this study we linked routine child health vaccination records to children’s MCS data to establish the timeliness of vaccine receipt in relation to recommendations in place at that time, with the objective of understanding the prevalence and distribution of delayed primary and pre-school vaccinations in a large nationally representative sample of children. Additionally, we compared parental report of their child’s MMR vaccination status with that routinely recorded in child health systems.

## 2 Methods

### 2.1 Study population

We used data from the MCS, a UK-wide nationally representative birth cohort comprising 18,818 children from 18,552 families born between September 2000 and January 2002. Parents were interviewed at home when their child was aged nine months and subsequently at three, five, seven, eleven and fourteen years of age. At the age seven home visit, written consent was sought from parents to link MCS information collected until each child’s 14th birthday, to data routinely collected by government departments or agencies, and other public sector organisations. The Northern and Yorkshire Research Ethics Committee gave approval for the MCS age seven survey; no additional approval was needed for this linked data analysis which focusses on those resident in Wales. Parents of 1840 (94.3%) of 1951 singletons resident in Wales, consented to health record linkage. Linked MCS and National Community Child Health Database (NCCHD) records were available for 1831 children. We excluded 46 children interviewed in countries other than Wales on one or more occasions by age 11 years and three for whom the main respondent was not the natural mother at the first interview, leaving a final sample of 1782.

### 2.2 Record linkage

We accessed coded data from the NCCHD, which brings together data from local child health system databases held by NHS organisations and includes information from birth registrations, child health examinations and immunisations.

We used the privacy-protecting Secure Anonymised Information Linkage (SAIL) Databank to store and access our data. Datasets imported into SAIL are anonymised and linked using a split file process preventing access to both identifiable data and clinical information at the same time. Records are linked through assigning unique encrypted Anonymised Linkage Fields (ALF) to person-based records [11].

### 2.3 Parental report of MMR vaccination

We compared parental response to the question “Has ^[cohort member] had any vaccination against measles, mumps or rubella (including MMR)?” asked at the age three interview, to NCCHD records of MMR vaccination, taking into account the age of the child at the interview and at MMR vaccination.

### 2.4 Timeliness of vaccinations

Vaccination schedules for the UK have changed repeatedly over the years. Children born in Wales between August 2000 and November 2001 should have received routine vaccinations as shown in Table 1. This cohort of children received separate DTP, polio, and Hib vaccines rather than the combination DTaP/IPV/Hib (5-in-1 vaccine) introduced in 2004. Although we considered analysing these vaccines as if they were a combination, a few children didn’t receive all the vaccines or received them on different dates, so we considered each vaccine separately.

**Table 1:** Vaccine schedule and definitions used for timeliness of vaccinations.

Vaccines	Vaccine doses due on the same occasion	Due at age	Timeliness of vaccination based on age at which vaccine received			
			Early	On time	Delayed	Never
Primary vaccines: Diphtheria, Tetanus and Pertussis *Haemophilus influenzae* type b Oral Polio Meningococcal group C	DTP 1, Hib 1, Polio 1, Men C 1	8 weeks	<8 weeks	8–12 weeks	>12 weeks	Not at all
	DTP 2, Hib 2, Polio 2, Men C 2	12 weeks	<12 weeks	12–16 weeks	>16 weeks	Not at all
	DTP 3, Hib 3, Polio 3, Men C 3	16 weeks	<16 weeks	16–20 weeks	>20 weeks	Not at all
Measles, Mumps and Rubella	MMR 1	1 year	<12 months	12–15 months	>15 months	Not at all
Pre-school boosters: Diphtheria, Tetanus and Pertussis Polio - oral or inactivated Measles, Mumps and Rubella	DTP PSB, Polio PSB, MMR 2	3 years, 4 months	<3 years, 4 months	3 years, 4 months to 5 years	>5 years	Not at all

Timeliness of vaccination was classified as early, on-time, delayed, or never, based on the recommended vaccination schedule. For the primary vaccines (diphtheria, tetanus, pertussis (DTP); oral polio vaccine; *Haemophilus influenzae* type b (Hib); Meningococcal C (Men C)) we defined a vaccine as being given ‘on time’ if given in the interval between the age when the vaccine was due and the age when the next dose was due; ‘early’ as being given prior to these ages; and ‘delay’ when given after the latest ‘on time’ ages (Table 1). For MMR and pre-school boosters, ‘on time’ was defined as 12–15 months and three years four months to five years respectively.

Child’s date of birth was supplied as week of birth (set to the Monday) and a day of birth within that week was assigned by adding a uniform random number between 0 and 6 days. Age at vaccination was calculated using this date and date of vaccination.

### 2.5 Statistical analysis

All analyses were performed using StataSE 13 (StataCorp. 2013. Stata Statistical Software: Release 13. College Station, TX: StataCorp LP.). Survey and non-response weights at age seven years were used to adjust for clustered sampling design, data missing due to losses to follow-up, and lack of consent to linkage. Weighted percentages were calculated and reported. Chi-squared tests were used to explore factors associated with delayed receipt of the first dose of DTP.

If there was uncertainty about receipt of a vaccine dose due to errors in the NCCHD records, participants (max *n* = 2) were omitted from the specific analysis involved.

## 3 Results

### 3.1 Characteristics of the cohort

We included 1782 children in this analysis (919; 51.6% boys). The majority (97.2%) was of a white ethnic origin. At the first MCS interview, 1233 (69.2%) lived in ‘disadvantaged’ and 549 in ‘advantaged’ electoral wards, with 1350 (75.8%) living in urban and 432 in rural areas. (Disadvantaged wards were oversampled [12] but the weights adjusted analyses making results nationally representative.)

### 3.2 Concordance between parental report and routinely recorded vaccination status

Parental report (99.1% mothers, 0.7% fathers, 0.1% grandmothers) of the first dose of MMR was compared with NCCHD MMR records. Taking into account when MMR vaccines were received in relation to the date of the MCS interview, at the time of the MCS age three interview, 89.8% of parental reports and 89.1% of NCCHD records (weighted percentages) suggested that children had received the first dose of MMR. The concordance between parental report and NCCHD record of MMR status was very high (97%) (Appendix 1).

In the MCS, when parents consulted the personal child health record (PCHR) to answer vaccination questions and information was available (*n* = 241), there were only three (1.2%) discrepancies between parental report of MMR status and the NCCHD record whereas when parents did not consult the PCHR (*n* = 1390), there were 45 (3.2%) discrepancies.

### 3.3 Timeliness of vaccinations

**Fig. 1:**
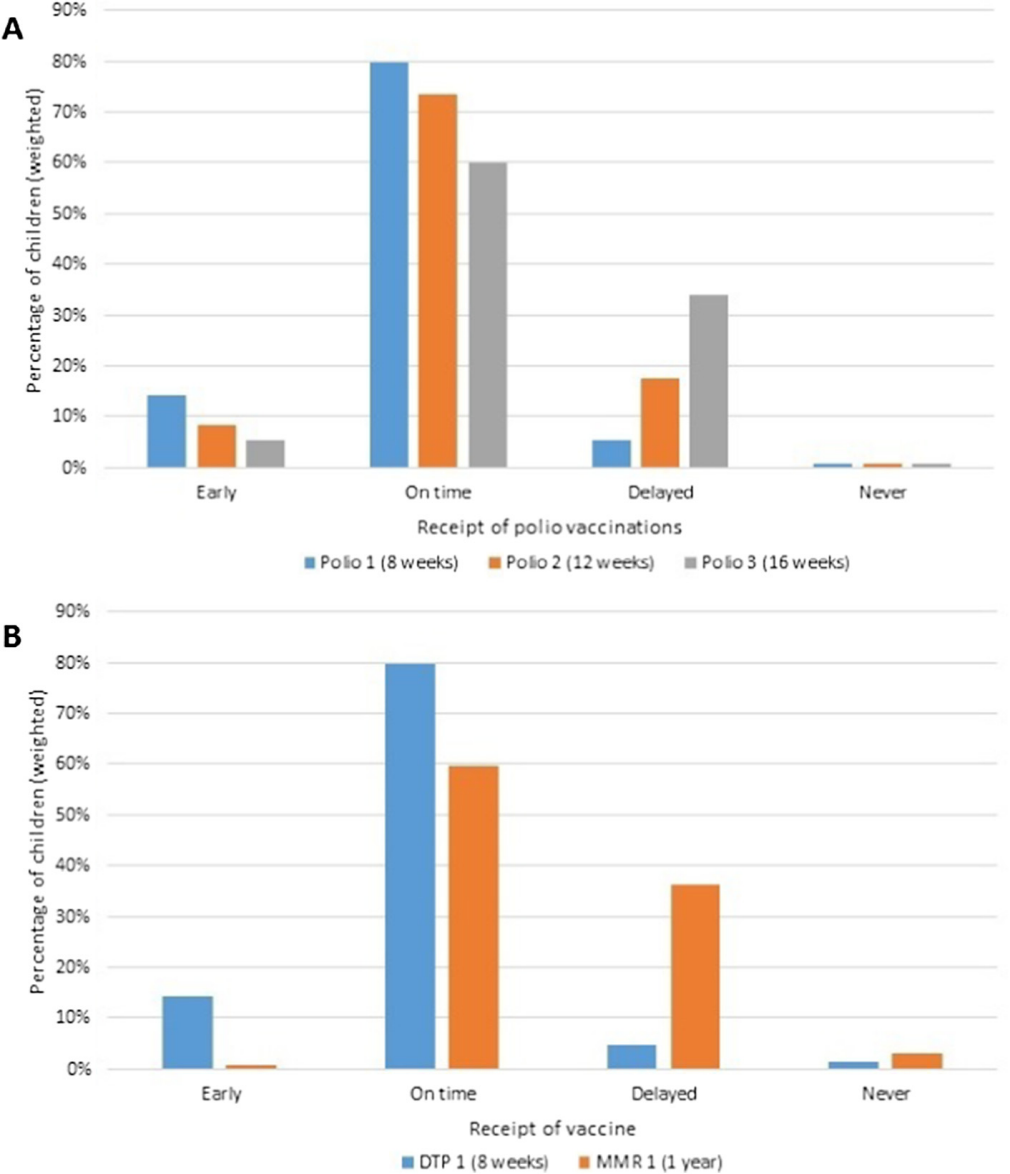
Timeliness of vaccinations (A) Polio vaccinations (B) DTP 1 and MMR 1 vaccinations.

The proportion receiving vaccines early or on time, decreased by dose as illustrated for polio (Fig. 1A); 79.6% of infants received the first dose on time but only 59.8% the third dose. By contrast, only 59.7% of children received the first dose of MMR on time compared with 79.7% for the first dose of DTP (Fig. 1B). Similar patterns were observed for the primary doses of Men C and Hib (Appendix 2).

Based on Department of Health guidance that the first doses of primary vaccines can be given from six weeks of age, very few children received vaccines so early as to be considered ineffective [13]. We found only four children received primary vaccinations before seven weeks of age.

**Fig. 2:**
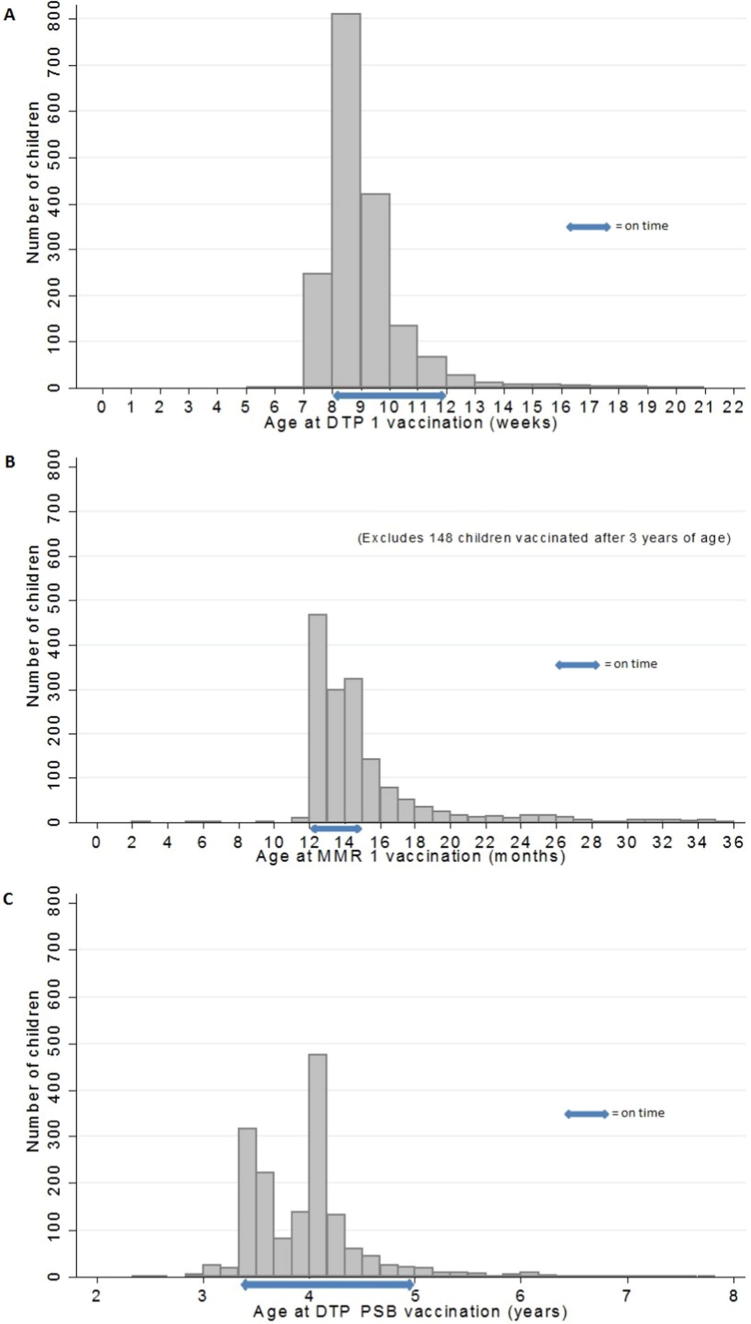
Age at vaccination (A) Age at DTP 1 vaccination (B) Age at MMR 1 vaccination (showing 0–3 years) (C) Age at DTP pre-school booster vaccination.

Delay in vaccination varied according to the vaccine and dose. For example, 30% of children with delayed DTP 1 were more than four weeks late (Fig. 2A), increasing to 38% and 47% for DTP 2 and DTP 3 respectively. Similarly for MMR 1, for 55% of those delayed, it was by more than three months (Fig. 2B), with some children (*n* = 51) not vaccinated until age 11 or 12 years, the majority during a measles outbreak. Pre-school boosters were more than six months late in two thirds of children with delay (Fig. 2C).

**Fig. 3:**
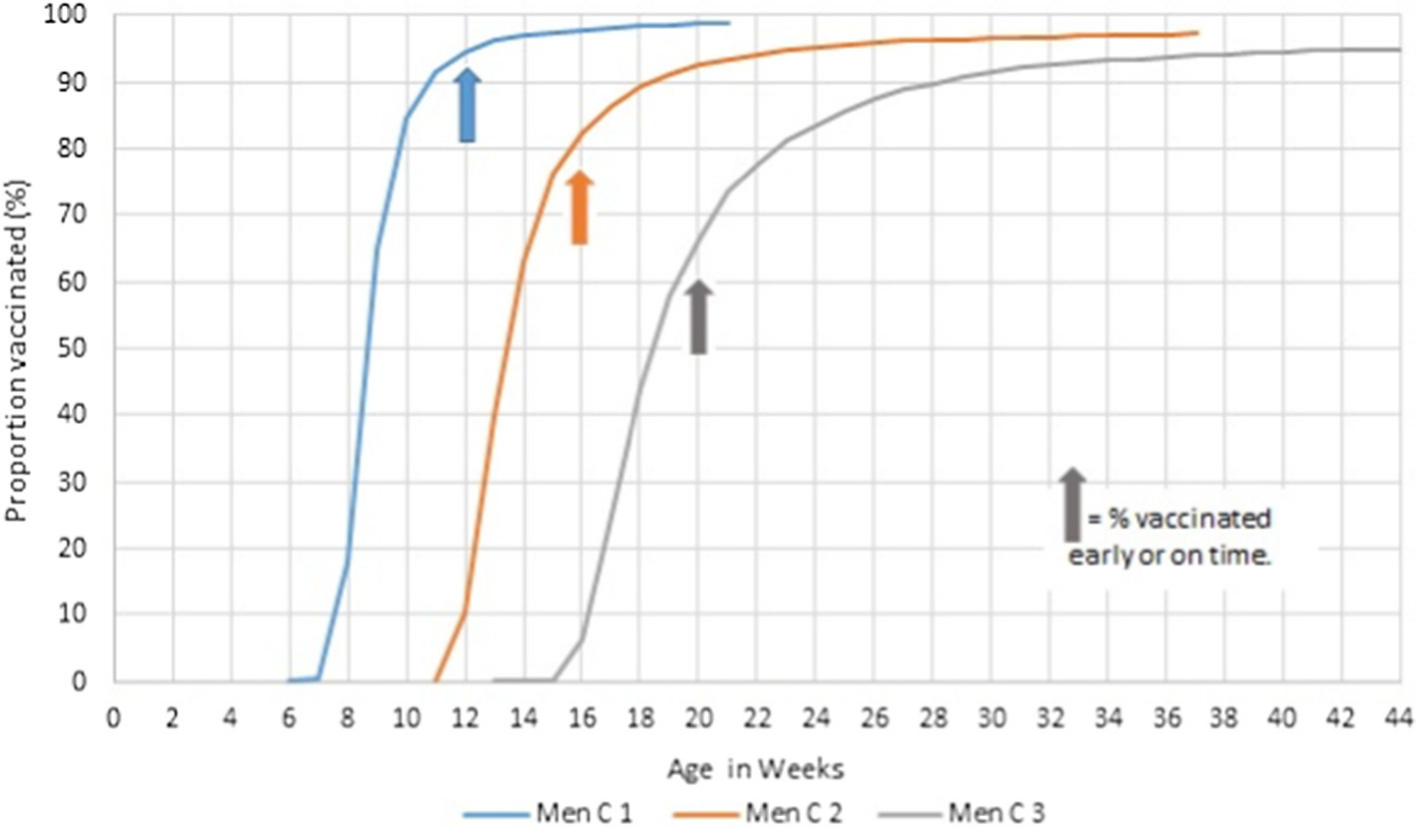
Age children received Men C vaccines.

Timeliness of Men C vaccination decreased with vaccine dose (Fig. 3), with 94%, 81% and 65% vaccinated early or on time (weighted percentages) for the first, second and third doses respectively (Appendix 2). Similar patterns were observed for DTP, polio and Hib (data not shown).

**Fig. 4:**
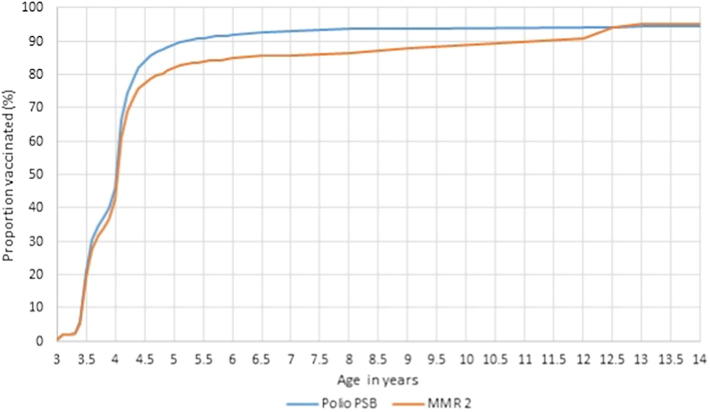
Age children received the pre-school booster vaccines.

Uptake of the second dose of MMR, although slightly lower, mirrored that of polio (Fig. 4). This shows that unlike the primary immunisations, pre-school boosters were not given uniformly over the period when vaccines were due. It suggests differing practices, with some immunised as soon as the vaccines were due whilst others were immunised at four years of age. Catch up of pre-school boosters continued throughout childhood, and eventually, uptake of the second dose of MMR equalled that of polio.

### 3.4 Time intervals between doses

**Fig. 5:**
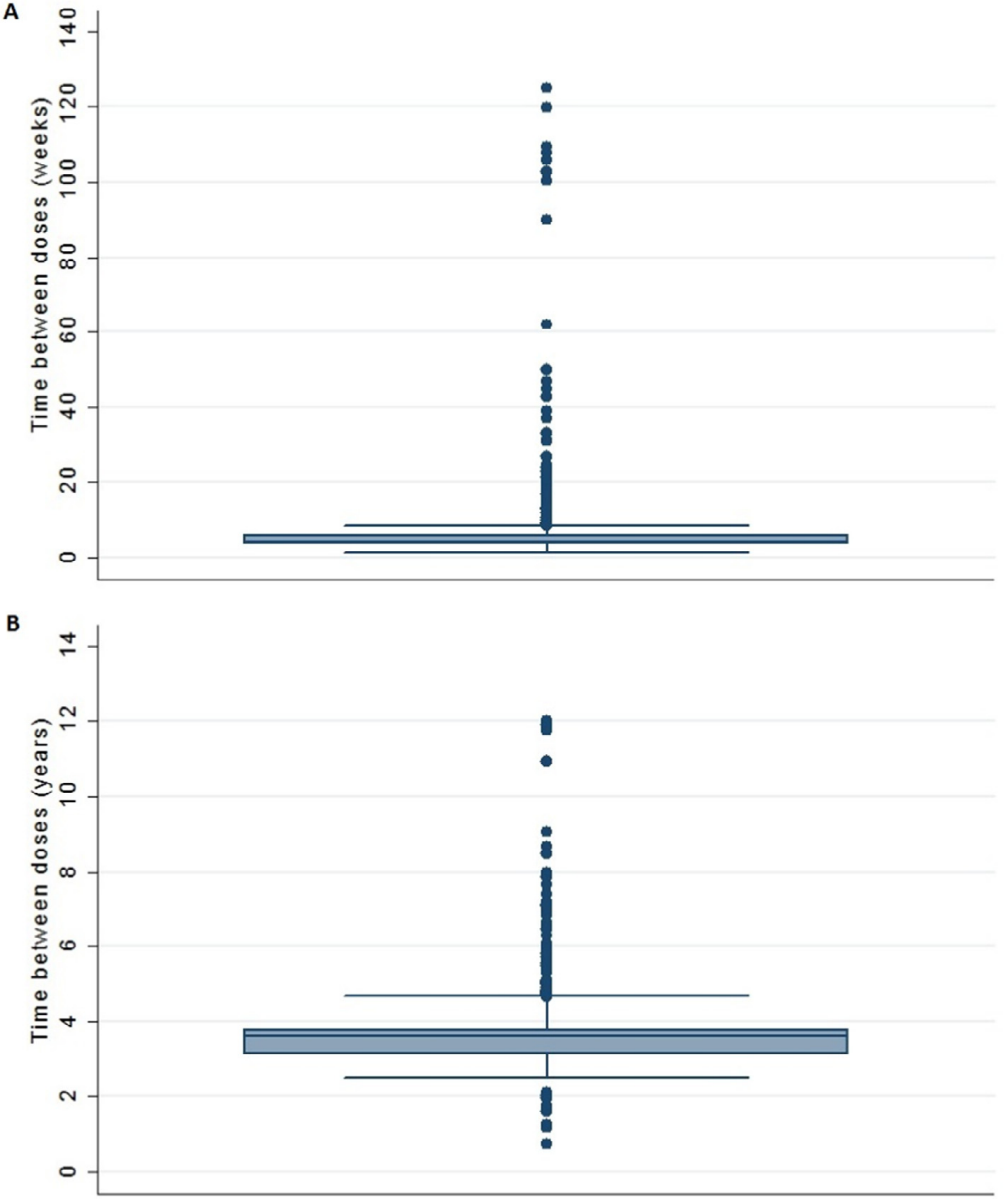
Boxplots of time between vaccine doses (A) weeks between DTP 1 and DTP 2 (recommended interval 4 weeks) (B) years between DTP 3 and DTP pre-school booster (recommended interval 3 years).

Although the median interval between the first and second doses was comparable to the recommended schedule, there was considerable variability between children in minimum and maximum intervals, with the median intervals between the second and third doses of the primary immunisations exceeding the recommendations (Fig. 5; Appendix 3).

### 3.5 Factors associated with delayed primary vaccinations

Boys (5.8%) were more likely than girls (3.7%) to have received the first dose of DTP late (*p* = .045). Delay of the first dose of DTP was also significantly associated with: larger family size (11.5% delayed where four or more children in the household, compared with 2.5% where only child in the household, *p* = .0001); lower maternal academic qualifications (8.9% of those whose mothers had no academic qualifications delayed compared with 1.2% of children whose mother had a degree, *p* = .01) and maternal unemployment (6.7% of those whose mother was not in work or on leave delayed compared with 3.0% where mother in work or on leave, *p* = .0016).

## 4 Discussion

This is the first UK study using linked cohort and routine immunisation data to explore uptake and timeliness of childhood vaccination in a nationally representative sample. Routine community child health data contain rich information on the timeliness of vaccination and we have demonstrated the completeness and use of this to understand the distribution of delayed primary and pre-school vaccinations in a large sample of children.

Uptake of the first doses of all the primary vaccines was high and similar with around 94% given early or on time (Appendix 2). However, timeliness decreased with the number of subsequent doses and timeliness of the first dose of MMR vaccine was notably worse than for other vaccines. Similarly, the proportion of children with delayed receipt of vaccines increased with subsequent doses, amplifying the risks associated with incomplete vaccination. These findings are consistent with those reported by Hull and McIntyre in Australia [2]. The proportion of children not vaccinated at all (up to the age of 14 years) ranged from 0.8% for the primary course of polio to 6.3% for the DTP pre-school booster. Delay of the primary vaccinations was associated with larger family size, lower maternal academic qualifications and maternal unemployment. This suggests delayed vaccination may be associated with deprivation in turn reflecting challenges accessing services in a timely manner due to other pressures.

Our finding that the timeliness of the first dose of MMR was poorer than that of other vaccines was unsurprising as these children were due to have their MMR vaccine in 2001–2, at the peak of public anxiety over the vaccine’s safety triggered by the now discredited 1998 Lancet publication [14]. By the time the second dose of MMR was given (2004 onwards) confidence appears to have improved; 80% received MMR 2 on time and during the period the vaccines were due, for any given age, uptake of MMR 2 was only a few percentage points lower than that for the pre-school boosters. COVER data also reflects the improvement in uptake of the second MMR dose by 2004 [15].

Any delay in receiving scheduled vaccines leaves children inadequately protected and at risk of infectious diseases compromising both their own protection and population immunity. We found that some children were at risk of potentially severe infections for many years. For example, 51 children (2.9%) only received the first dose of MMR when they were aged 11 or 12 years, and for the majority of these, this appeared to be in response to a large measles outbreak in Wales between November 2012 and July 2013 [16].

The strengths of this study are that it was based on a nationally representative sample, used routine health records with a high proportion of parents consenting to record linkage and a high proportion of records were successfully linked. The completeness of NCCHD records and the triangulation with parental report showing agreement demonstrates good quality data in routine records and increased the validity of our findings.

This cohort of children received separate DTP, polio, and Hib vaccines rather than the 5-in-1 vaccine introduced in 2004 or the 6-in-1 vaccine introduced in 2017. Whilst the majority of children received the separate vaccines on the same occasion as intended and parents did not omit or delay specific vaccines, we would still anticipate improved vaccine uptake since the introduction of the combination vaccines.

We were not able to calculate exact ages at vaccination as day of birth was withheld to minimise risk of disclosure. As birth day was allocated randomly this should not have affected the proportions of vaccines estimated to be early, on-time or delayed.

Overall the quality of the NCCHD records was good, an important requirement for effective monitoring of vaccine uptake [17]. Errors in NCCHD vaccination records were identified for 40 (2.2%) children, and were mainly due to recording two doses of MMR 2 and none for MMR 1, perhaps reflecting confusion due to the vaccine trade name MMR® II, or that when a child has their first MMR vaccine at an older age it is coded as the second instead of the first. Additionally, we questioned the accuracy of some data which may or may not have been data entry errors. For example, doses given very early or with a reduced gap may represent a data entry error, atypical practice or atypical circumstances.

Tickner’s study of parental views about pre-school immunisation, reported some found it harder taking older and thus more aware children for vaccination. Parents also reported less contact with health visitors when pre-school boosters and second MMR doses are due and the lack of information provided to them, made them question their importance [18]. Although, immunisation reminders are highly effective at increasing uptake [19] parents may also need a more personalised approach to be reminded of the continued importance of immunisation in older children. Recently, an increase in use of alternative vaccine schedules (intentional deviation from the routine schedule) has been reported from the USA which include delay in commencing, rejection of some vaccines and spacing of others [20]. We suspect this is unlikely to have been a significant factor in this population, but as estimation of vaccination timeliness in UK is not routinely conducted, this cannot currently be monitored.

Previous studies of immunisation uptake from the same cohort and based on parental report suggested high immunisation uptake in the cohort participants. Using data from the first interview at age nine months, Samad et al. reported that 95.6% of all MCS infants were fully immunised [8]. Our data showed that at nine months (39 weeks) of age, 94% of Welsh infants had received three doses of DTP, Men C, Hib and polio according to NCCHD records. Similarly, using data from the age three MCS interview, Pearce et al. reported that 88.6% of participants had received MMR vaccine (10, 21) this compares with our finding that 88.2% of Welsh participants received their first dose of MMR by three years of age according to NCCHD records. While these earlier studies included the whole UK MCS population (and differences were noted between countries), and participants were not exactly nine months or three years of age at the interviews, uptake rates based on parental report and on health service data are similar. Additionally we demonstrated high levels of concordance between parental report and health records, of MMR immunisation status with even better agreement when parents refer to the PCHR. Where there are discrepancies, errors may lie in either parental recall or in the entries in the health records [22]. In contrast with the findings of a recent systematic review [21], we found parental report of immunisation uptake to be reliable.

Hull and McIntyre [2] propose that with high vaccine coverage achieved in most industrialised countries, vaccination timeliness is the “next benchmark to aim for in programme performance”. Although our analysis using routine data in Wales is not based on a current cohort of children and hence estimates of timeliness may not reflect current practice, we have demonstrated the value of exploring this measure. Although Scotland publishes timeliness of primary and MMR vaccines by deprivation [23], it is not easy to conduct the same analysis for the whole of the UK as England does not currently have a unified child health database. We are conducting further work based on the linked MCS and NCCHD records to investigate further those factors associated with vaccine delay to inform policy and practice. Timely immunisation is important to provide children with maximum protection against serious infectious diseases. We have demonstrated the feasibility and value of using routine child health data to explore vaccine timeliness and recommend that in addition to vaccine coverage, a measure of vaccination timeliness is used routinely to evaluate the quality of immunisation services.
