# Supplementary material for: Measuring the timeliness of childhood vaccinations: Using cohort data and routine health records to evaluate quality of immunisation services
Source: Vaccine. 2017 Dec 18;35(51):7166–73. doi: 10.1016/j.vaccine.2017.10.085 (PMC5720480; doi:10.1016/j.vaccine.2017.10.085)
Supplement: Supplementary data 1 [file mmc1.docx]

**APPENDIX 1 – Concordance between parental report and NCCHD records for first dose of MMR**

| **APPENDIX 1** | | NCCHD record of MMR 1 (at time of MCS2) | |  |
| --- | --- | --- | --- | --- |
|  |  | Yes | No | Total |
| Parental report of MMR 1  (at MCS2) | Yes | 1435 | 28 | 1463 |
|  | No | 22 | 160 | 182 |
|  | Total | 1457 | 188 | 1645 |

**APPENDIX 2 – Timeliness of vaccines (weighted percentages)**

| **Vaccine Dose** | **Early** | **On time** | **Delayed** | **Never** |
| --- | --- | --- | --- | --- |
| Polio 1 (8 weeks) | 14.1% | 79.6% | 5.5% | 0.8% |
| Polio 2 (12 weeks) | 8.3% | 73.5% | 17.4% | 0.8% |
| Polio 3 (16 weeks) | 5.4% | 59.8% | 34.0% | 0.8% |
|  |  |  |  |  |
| DTP 1 (8 weeks) * | 14.2% | 79.7% | 4.8% | 1.4% |
| DTP 2 (12 weeks) * | 8.2% | 73.6% | 16.5% | 1.6% |
| DTP 3 (16 weeks) * | 5.5% | 60.1% | 33.0% | 1.5% |
|  |  |  |  |  |
| Men C 1 (8 weeks) | 14.0% | 79.7% | 5.3% | 1.1% |
| Men C 2 (12 weeks) | 8.2% | 73.2% | 17.2% | 1.5% |
| Men C 3 (16 weeks) | 5.5% | 59.4% | 33.1% | 2.0% |
|  |  |  |  |  |
| Hib 1 (8 weeks) | 14.2% | 80.0% | 5.0% | 0.9% |
| Hib 2 (12 weeks) | 8.2% | 73.7% | 17.1% | 1.0% |
| Hib 3 (16 weeks) | 5.5% | 60.0% | 33.6% | 1.0% |
|  |  |  |  |  |
| MMR 1 (1 year) | 0.8% | 59.7% | 36.3% | 3.2% |
|  |  |  |  |  |
| Polio PSB (3 yr 4m) | 2.6% | 86.2% | 5.4% | 5.8% |
| DTP PSB (3 yr 4m) ** | 2.5% | 85.7% | 5.4% | 6.3% |
| MMR 2 (3 yr 4m) | 2.3% | 80.0% | 12.7% | 5.0% |
|  |  |  |  |  |
|  |  |  |  |  |
| * A further 10 children had DT 1, 14 had DT 2 and 14 had DT 3 | | | | |
| ** A further 12 children had DT | | | | |

**APPENDIX 3 - Time intervals between doses**

| **Vaccines** | **Recommended** | **Time intervals between doses** | | | |
| --- | --- | --- | --- | --- | --- |
|  | **intervals *** | median | mean** | minimum | maximum |
|  |  |  |  |  |  |
| Polio 1 and Polio 2 | 4 weeks (28 days) | 29 days | 42 days | 10 days | 2.40 years |
| Polio 2 and Polio 3 | 4 weeks (28 days) | 35 days | 54 days | 6 days | 12.03 years |
| Polio 3 and Polio PSB | 3 years | 3.60 years | 3.62 years | 277 days | 12.04 years |
|  |  |  |  |  |  |
| DPT 1 and DPT 2 | 4 weeks (28 days) | 29 days | 42 days | 10 days | 2.40 years |
| DPT 2 and DPT 3 | 4 weeks (28 days) | 35 days | 53 days | 6 days | 8.86 years |
| DPT 3 and DPT PSB | 3 years | 3.59 years | 3.62 years | 277 days | 12.04 years |
|  |  |  |  |  |  |
| Men C 1 and Men C 2 | 4 weeks (28 days) | 29 days | 45 days | 10 days | 9.82 years |
| Men C 2 and Men C 3 | 4 weeks (28 days) | 35 days | 51 days | 6 days | 5.29 years |
|  |  |  |  |  |  |
| Hib 1 and Hib 2 | 4 weeks (28 days) | 29 days | 43 days | 10 days | 2.40 years |
| Hib 2 and Hib 3 | 4 weeks (28 days) | 35 days | 53 days | 6 days | 8.86 years |
|  |  |  |  |  |  |
| MMR 1 and MMR 2 | 2 years 4 months (2.33 years) | 2.72 years | 2.96 years | 28 days | 12.09 years |
|  |  |  |  |  |  |
|  |  |  |  |  |  |
| * If both vaccines given at the scheduled times | | |  |  |  |
| ** weighted |  |  |  |  |  |
